# Scoping Review of Outdoor and Land-Based Prevention Programs for Indigenous Youth in the United States and Canada

**DOI:** 10.3390/ijerph22020183

**Published:** 2025-01-28

**Authors:** Faith M. Price, Tara D. Weaselhead-Running Crane, Elizabeth H. Weybright

**Affiliations:** 1 Department of Human Development, Washington State University, Pullman, WA 99164, USA; elizabeth.weybright@wsu.edu; 2 Center for Indigenous Health, Bloomberg School of Public Health, Johns Hopkins University, Baltimore, MD 21205, USA; 3 School of Speech, Language, Hearing & Occupational Sciences, College of Health, University of Montana, Missoula, MT 59812, USA; 4 Department of Psychology, University of Montana, Missoula, MT 59812, USA

**Keywords:** Indigenous, land-based, outdoor, prevention, Indigenous wellbeing, Native American

## Abstract

Interventions taking place on the land are culturally well aligned for Native peoples, as they are often developed by the community and incorporate traditional knowledge, values, and practices. However, research on the effectiveness and characteristics of such programs is lacking. This scoping review examined outdoor and land-based prevention interventions for Indigenous adolescents ages 10–25 in the United States and Canada to identify program characteristics such as origination, aims, activities, duration, evaluation methods, and outcomes. Over three-fourths (77%) of the 153 programs identified were community-derived. The programs were principally strength-based and promoted protective factors for general wellbeing. The most common delivery format was short camps. Nearly all programs (97%) included an element of culture. The activities most often seen were recreation (84%), subsistence living (65%), and Elder knowledge sharing (63%). Thirty-three studies measured outcomes and included quantitative, qualitative, and mixed method study designs. Studies found positive impacts on participants’ self-esteem and mental health; connections to culture, cultural pride, and identity; and connections to community including peers and Elders. The literature on outdoor and land-based prevention interventions for Indigenous youth is growing rapidly. Understanding program components is a first step to identifying the elements critical to effective programs for Indigenous youth.

## 1. Introduction

An increasing number of Indigenous communities are implementing programs aimed at undoing the damage of the colonial past through renewal of cultural lifeways [1]. Since colonization was “fundamentally about dispossessing Indigenous people from the land, decolonization must involve forms of education that reconnect Indigenous peoples to land and the social relations, knowledges and languages that arise from the land”, [2] (p. i). Reconnecting to the land is seen as part of the necessary healing process from the traumas of colonization. Moreso, land itself is seen as a healer, as the foundation of culture and an integral part of Creation, of which we are all a part of [3,4]. Prevention programs that include outdoor settings therefore are a natural fit for Indigenous communities.

There is considerable literature documenting the mental, physical, and social health benefits of nature-based programs in the general population, often referred to as “outdoor” or “adventure” programs [5,6,7]. Outward Bound is one of the most well-known outdoor adventure programs, largely credited with developing the model for such programs [8]. In general, outdoor programs facilitate group problem solving and decision making around challenging experiential activities in a natural environment [6,9,10]. The outdoor setting offers natural challenges that produce feelings of mastery, competence, and positive self-concept in a venue that, unlike the manmade environment, reduces stress and enhances mood [11].

Outdoor adventure-based programs have been utilized in Indigenous communities; however, culturally based outdoor programs are more common. Prevention programs that are grounded in the culture of their intended population are more likely to be relevant and acceptable within that community, and ultimately find more success [12]. Grassroots programs that include traditional knowledge and cultural practices taking place on the land and waterways have developed throughout Indigenous communities in the United States and Canada [13,14,15]. In Canada, they have commonly been termed “land-based” programs. Land-based programs are culturally well aligned for Indigenous peoples, as they are typically developed by the community, and incorporate traditional knowledge, values, and practices. As such, they are a socially and culturally acceptable mode of providing health and wellness programming to Indigenous communities [4,14]. In addition, they promote Indigenous self-determination through strengthening and/or renewal of cultural values, ethics, relationships, and life skills [3].

The United States and Canada’s shared history of damaging colonial policies and practices has left Indigenous youth in both countries at elevated risk for substance use and poor mental health outcomes. Native American youth in the United States have the highest rates of suicide of any racial/ethnic group [16]. Native American youth begin using substances earlier than other populations [17] and have some of the highest rates of substance use disorder of any other racial/ethnic group between the ages 12 and 25 [18]. In Canada, suicide rates [19] and substance use rates [20] for First Nations, Inuit, and Métis youth and young adults are also many times higher than for non-Indigenous youth and young adults. First Nations Canadian youth ages 15–24 are six times more likely to die by suicide than their non-Indigenous counterparts [19]. Canadian Indigenous youth are also three times as likely to have tried marijuana and five times as likely to smoke tobacco [20]. There is a need for more effective, culturally appropriate prevention approaches for the Indigenous youth population in light of these disparities [12,15].

In the past, prevention efforts with Indigenous communities have been based on theories and frameworks developed from Western thinking that may not resonate with or perhaps even conflict with Indigenous ways of knowing [15]. Factors such as culture, spirituality, and community that are integral to Indigenous lifeways are disregarded by more individual-centered theories of change [21]. Prevention programs based on the non-Indigenous experience may even be viewed as an extension of colonization [22,23]. More evidence-based programs designed by and for Indigenous populations are needed, as adaptations of evidence-based programs from non-Indigenous cultures are inadequate [1].

Numerous Indigenous communities have implemented grassroots prevention efforts based on traditional knowledge and cultural protective factors that prevention researchers could learn from, but they are largely absent in the scientific literature [15]. Although there is a growing number of culturally grounded programs developed for Indigenous youth, the publication of data on their effectiveness has lagged for a number of reasons [12,24]. Tensions exist between Indigenous communities and outside researchers, due to historical unethical treatment and devaluing of Indigenous knowledge [25,26]. In addition, instruments to evaluate program outcomes are not commonly developed for or tested on Indigenous people and therefore may lack cultural validity [27,28]. Tribes have oftentimes opted to implement “Tribal Best Practices” that have cultural approval but may lack published research of their effectiveness [13,15]. Increasingly, however, Indigenous knowledge, theories, and approaches are being confirmed by research and published in the scientific literature [15].

While grassroots land-based and other outdoor youth programs are culturally acceptable in Indigenous communities, there remains a dearth of research on how to design, implement, or evaluate them [14]. One early study of outdoor programs in Indigenous contexts examined lessons learned from an adaptation of Outward Bound to an Indigenous Canadian community [29]. Findings indicated that the curriculum needed to be grounded in the culture and traditions of the community, much as land-based programs have achieved. More recently, the Thunderbird Partnership Foundation [3] reviewed eight land-based programs to develop a model of land-based health services as a guide for communities. Two of the programs they assessed were youth prevention programs. Some common components of those two programs included a foundation of cultural values, teaching of traditional living skills, and an environment that fostered participant and community connections.

The body of knowledge on prevention programs designed for Indigenous communities is growing; however, in addition to peer-reviewed articles, it may be in unpublished evaluations or reports, referred to as the “grey literature”. The literature available on land-based programs has grown tremendously in the past few years, and outcomes show promise such as positive impacts on mental health [30]. Land-based programs have been used all along the continuum of care, from health promotion and prevention to treatment [3], and have emerged as an increasingly common approach in recent reviews of prevention programs in Indigenous communities [31,32,33]. One review of the literature specifically on land-based physical activities for Indigenous adults identified nine studies with qualitative findings related to mental, emotional, social, cultural, and physical benefits of involvement [34]. Participants reported deeper cultural and community connections, increased physical activity, improved nutrition, better coping skills, and increased social support. To our knowledge, no broad, systematic review of land-based or outdoor prevention programs for youth has been published.

We conducted a scoping review of outdoor and land-based prevention programs for Indigenous youth to assess program characteristics and evaluation. The current study examined the breadth of the available literature on outdoor and land-based prevention programs for Indigenous youth in the United States and Canada to answer the following research questions:

1. What are the characteristics (origination, target population, aims, duration, activities) of outdoor and land-based prevention programs for Indigenous youth?
2. Among the programs evaluated, what were the (a) methodologies used, (b) specific outcomes assessed, (c) measurement tools or instruments used, and (d) findings?

A scoping review is a method used to provide a broad overview of the available literature in a research area [35] and is commonly used when trying to establish a knowledge base, map types of evidence available, find gaps in the literature, clarify concepts, and/or explore whether to pursue a systematic review [35,36,37]. They are helpful in assessing the evidence in an emerging area and include both published studies and other literature [36]. This is especially useful for a topic such as Indigenous outdoor and land-based programs, many of which are grassroots efforts that may not be published in a scientific journal.

Land-based programs are a rapidly growing and, until recently, little-documented prevention program type. An initial step in understanding the utility of outdoor and land-based programs would be to see the characteristics of programs that exist and why they are viewed as effective [15].

## 2. Materials and Methods

Scoping review methodology comprises five required steps: (1) identifying the research question; (2) identifying relevant studies; (3) study selection; (4) charting the data; (5) collating, summarizing, and reporting the results [35]. This scoping review is organized by these steps and followed the Joanna Briggs Institute (JBI) guidelines for scoping reviews [36] and the Preferred Reporting Items for Systematic Reviews and Meta-Analyses–Extension for Scoping Reviews (PRISMA-ScR) [38]. The complete protocol for this review was made available online through the Open Science Framework (OSF Registration: https://doi.org/10.17605/OSF.IO/62BMC, accessed on 23 January 2023).

### 2.1. Identifying the Research Questions

The intent of this review was to identify the individual programs mentioned within the literature to gauge how widespread outdoor and land-based programs are and identify key characteristics to, long-term, inform best practices in common. To that end, this scoping review asked Research Question 1, “What are the characteristics (origination, target population, aims, duration, activities) of outdoor and land-based prevention programs for Indigenous youth?” Secondly, there is a lack of documented evidence on outdoor and land-based prevention programs for Indigenous youth, due in part to evaluation methods that conflict with Indigenous ways of knowing and instruments that have not been tested on or validated with Indigenous communities [13,15,28]. This led to Research Question 2, “Among the programs evaluated, what were the (a) methodologies used, (b) specific outcomes assessed, (c) measurement tools or instruments used, and (d) findings?”

### 2.2. Identifying Relevant Studies

#### 2.2.1. Eligibility Criteria

Peer-reviewed journal articles and the grey literature (e.g., dissertations, reports, websites) written in English and published between the period of 1 January 2000 and 30 November 2023, which met the following inclusion criteria, were included in this review.

- The program described must include activities in a natural setting. Both mainstream outdoor adventure programs and land-based cultural programs were included.
- The program had to focus on prevention or wellness promotion, with a mental, emotional, or behavioral health aim. Programs that focused solely on physical health (i.e., diabetes or obesity prevention, physical activity promotion) were not included.
- The program must serve Indigenous youth, including emerging adults, ages 10–25 years old.
- The program must be located in the United States or Canada.

#### 2.2.2. Info Sources

Eight electronic databases and four key websites were searched between October 2022 and November 2023. Databases included: PsycINFO, PubMed, Web of Science, Cumulated Index to Nursing and Allied Health Literature (CINAHL), Indigenous Studies Portal, Native Health Database, Circumpolar Health Bibliographic Database, and Arctic Health Publications Database. Manual searches of the following websites were conducted: The Health Council of Canada: Aboriginal Health, Public Safety Canada, the National Indian Health Board, and the Indian Health Service. Due to the lack of a search function, The Center for American Indian and Alaska Native Health and The National Collaborating Centre for Aboriginal Health websites were not included.

#### 2.2.3. Search Strategy

The following keywords were used in various combinations: youth, adolescent, young adult, emerging adult; Native American, American Indian, Alaska Native, Native Hawaiian, Tribal, Indigenous, Aboriginal, First Nation; land-based, outdoor, on-the-land, adventure, wilderness, nature-based, recreation, cultur*, equine; health, wellness, mental health, substance abuse, substance use, suicide, depression; program, intervention, prevention, camp; evaluat*, outcome, impact, and effectiveness. The complete search strategy for PubMed is included as an example in the Supplementary Materials, Table S1.

### 2.3. Study Selection

**Figure 1:**
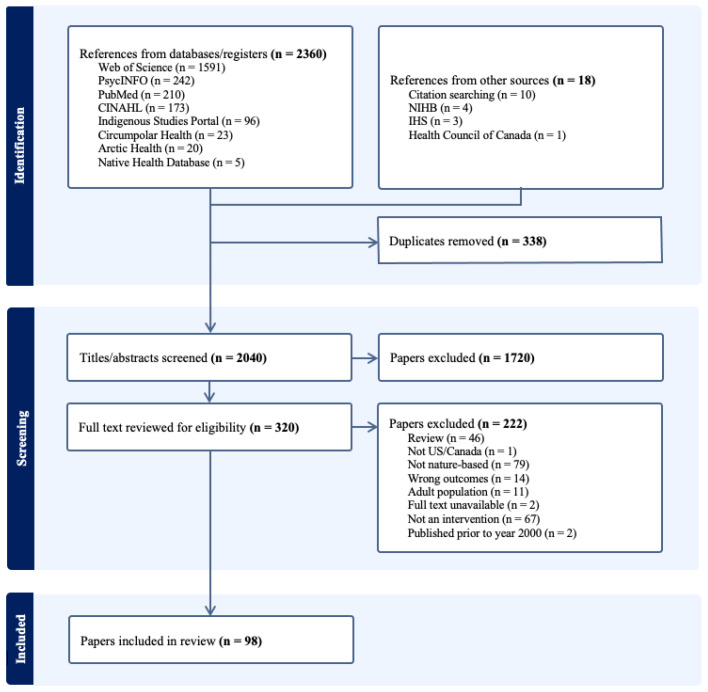
PRISMA flow chart detailing search and study selection.

Search results of the peer-reviewed and grey literature were imported into Covidence, systematic review software. Initially, 2360 references were identified from databases and registries, and another 18 from websites and citation searches. After removing 338 duplicates, 2040 document titles and abstracts were screened by the first and second authors. After screening, 1720 references did not meet the inclusion criteria and were excluded. The full texts of 320 remaining publications were retrieved and screened by the research team. An additional 222 references did not meet inclusion criteria and were excluded, leaving a total of 98 documents included in the review. See Figure 1 for a flow chart of the search, inclusion, and exclusion process. The first and second authors met regularly to discuss progress and resolve inconsistencies in reviewer assessment through a consensus.

### 2.4. Charting the Data

A data extraction form was developed in Covidence, tested, revised, and used to inventory 153 unique programs across the 98 documents (some documents described multiple programs; see Supplementary Materials Table S2). Data collected from the documents included: the author(s), year of publication, location (tribal community, state/province, country), target group, program type (prevention/promotion), program origination/development (adaptation or community-derived), duration of the program, program aims, program components (activities), specific land-based activities, evaluation methodology (if applicable), outcome measures, and reported outcomes. Data were exported from Covidence into Microsoft Excel for the analysis.

### 2.5. Collating, Summarizing, and Reporting the Results

Program characteristics and evaluation methods were analyzed using frequencies and a thematic analysis. The data extraction fields were filtered in Excel and the number of programs fitting each characteristic was tabulated. Within the Aims, Intervention Components, and Land-Based/Outdoor Activities fields where answers were open-ended, the data were reviewed for similarities, and themes were identified by the authors. The filter function was then used to search based on relevant words per theme and tabulate the number of programs identified as having that characteristic.

## 3. Results

### 3.1. A Description of the Included Literature

A total of 98 documents describing programs for Indigenous youth and/or young adults with outdoor or land-based elements were identified. Fifty-one of these documents were peer-reviewed articles and 47 came from the grey literature (e.g., government reports, websites, newsletters). The amount of the available literature on outdoor and land-based programs has been growing rapidly, with most of the documents (*n* = 68, 69%) published in the past decade (2013–2023). Table 1 provides a breakdown of the included literature by types of documents. Most of the literature came from Canada (*n* = 61; 62%) including over three-fourths of the grey literature (36 of 47; 77%) and 49% of the peer-reviewed literature (*n* = 25).

### 3.2. Program Characteristics

A total of 153 unique programs were identified in the literature. The majority of programs (*n* = 120, 78%) were located in Canada, while 33 (22%) were in the United States. Most programs (*n* = 104; 68%) solely served youth under age 18; however, 3% (*n* = 5) of programs served young adults and 8% (*n* = 13) were inclusive of both youth and young adults. Another 20% (*n* = 31) included youth and their families and/or community members as the target population.

Many programs (118 of 153, 77%) were developed in a grassroots fashion by the communities they were implemented in. An additional 16 (10%) were developed by the community in collaboration with researchers. Thirteen programs were adaptations (8%) of other programs either created for the general population or designed by and for Indigenous peoples and adapted to other Indigenous communities. Three (2%) were developed by outside agencies and implemented without adaptation, and three (2%) were unclear in how they originated.

Most of the programs (*n* = 109; 71%) were designed to promote general mental wellness; however, some focused on substance use prevention (*n* = 22; 14%), suicide prevention (*n* = 12; 8%), or substance use and suicide prevention in combination (*n* = 10; 7%). Many programs (*n* = 54; 38%) solely articulated general aims of the prevention of substance use and suicide or promotion of wellbeing. However, 99 (65%) identified risk or protective factors they expected their programming to impact. The programs were largely strength-based and focused on promoting protective factors for their youth participants. Among these 99 programs, five common themes emerged: personal development, connection to culture, connection to community, leadership, and connection to the environment. Aspects of personal development were named by 55 programs (56%) and broadly encompassed healing, resilience, coping skills, self-esteem, self-efficacy, purpose, and hope. The second most common theme was to increase connection to culture (*n* = 49; 49%), which included desires for youth to grow in their cultural knowledge, practices, language, and history, and the promotion of positive cultural identity. Connection to community (*n* = 24; 24%) included developing healthy relationships with peers, parents and families, adults, role models, Elders, and the community overall, as well as building a sense of belonging. Ten programs (10%) aimed to increase youth leadership skills and nine programs (9%) specified a desire to connect youth with the environment, including land and water, as an important relationship, and source of healing, and for its important cultural and historical context.

Program durations ranged from 1-day outdoor prevention events (*n* = 4; 3%), to once/year camps up to one-week long (*n* = 33; 22%), to multiple seasonal short camps or events (*n* = 62; 41%), to more substantial 9-day-plus camps and full-year programs (*n* = 54; 35%).

### 3.3. Program Activities

As the literature was screened, common program activities began to emerge. Our data extraction tool evolved to include the following components: subsistence living skills; recreation; equine experience; community events and gatherings; Elder knowledge sharing; spiritual practices; celebrations and feasts; talking circles or group discussions; arts and crafts; community service; formal curriculum and/or lessons; and cultural activities, unspecified. We also included an ‘other’ category of unique items. See Table 2 for an overview of the number of programs found incorporating each activity.

#### 3.3.1. Culture

Nearly all (*n* = 149; 97%) of the 153 programs featured culture. This became apparent during the thematic analysis when examining characteristics in combination with those that were culturally based such as subsistence living skills, Elder knowledge sharing, spiritual practices, significant sites, Indigenous language, values, and unspecified cultural activities. Only four programs (3%) offered outdoor activities exclusive of cultural elements. These included a skiing program, a snowboarding program, a walking intervention, and a program that offered wilderness trips. Except for a one-day educational walking intervention, recreation-based programs sans culture were developed outside of the communities they served.

#### 3.3.2. Recreation

Many of the programs involved recreation (*n* = 128; 84%), including games, sports, and activities more commonly viewed as outdoor recreation (e.g., camping, canoeing, rock climbing). This category included activities even when its primary purpose may have been a mode of transportation (e.g., boat journey, snowmobiling, walk) as well as camping, although the intent may have been to practice a traditional lifeway rather than to recreate per se. The goal was to capture how many programs practiced these components regardless of its purpose. It is also notable that many traditional land- and water-based practices and lifeways bring the same pleasure as ‘recreation’ endeavors today.

Most programs that offered recreation (*n* = 124; 97%) provided either a cultural form of recreation (e.g., camping, canoeing, snowshoeing, traditional games) or recreation in combination with cultural activities, knowledge, or traditional subsistence living skills. For example, the Outdoor Adventure Leadership Experience in Ontario, Canada, has participants travel by canoe, a traditional transportation method for their Anishnaabe community, on a 10-day journey through their homelands and significant sites [28]. In contrast, the Reducing Risk through Interpersonal Development, Empowerment, Resiliency, and Self-Determination (RezRIDERS) program based in Jemez Pueblo in New Mexico features outdoor recreation activities that are more contemporary (snowboarding, white-water rafting, high-ropes course, and rock climbing) but includes locations of cultural significance [39].

#### 3.3.3. Subsistence Living Skills

The third most common activity, subsistence living, was included in 100 (65%) programs. Subsistence living was defined as activities that promoted living off the land such as acquiring food (e.g., hunting, fishing, gathering), building shelter, survival skills, and traditional ecological knowledge such as plant medicines. The subsistence activities described were as diverse as the Tribes, lands, and waters they reside upon. For example, one community in Nunavut, Canada, had experienced hunters teach youth and young adults hunting, fishing, and seafood-gathering techniques, including navigation and traditional weather forecasting [40]. They also worked together to construct igloos. The Milo Pimatisiwin Project of Moose Cree First Nation described seasonal, one-week culture camps including “Fish Week”, “Spring Hunt Week”, and “Moose Week” where youth learned how to fish with nets and rods, snare rabbits, hunt moose, and prepare traditional Cree food [41].

#### 3.3.4. Elder Knowledge Sharing

Most programs (*n* = 97; 63%) included intergenerational learning, largely through Elders sharing knowledge with youth participants. What we termed “Elder knowledge-sharing” included Elders as well as cultural knowledge keepers (e.g., hunters, trappers, cultural experts). Many programs described youth and Elders going out on the land together for instruction in traditional living skills. The Omushkego Cree community in Ontario, Canada, sponsored multiple programs aimed at reconnecting youth with Elders, the land, and cultural traditions by employing Elders and other cultural experts to lead youth in revitalizing traditional harvests of beaver, goose, and fish [42,43,44]. One aspect of the Smart Indigenous Youth program in Saskatchewan, Canada, involved taking youth on “medicine walks” with Elders and cultural knowledge keepers to gather sacred plant medicines [45].

#### 3.3.5. Arts and Crafts

Arts and crafts were inclusive of contemporary and traditional visual and expressive arts. In total, 54 of the 153 programs (35%) included an arts and crafts element. The specific types of arts and crafts were often not mentioned, nor were they extracted, unless it took place on the land. For example, a program that included hunting and hide processing that also included drum making was recorded as arts and crafts [46].

#### 3.3.6. Community Events and Gatherings

We identified 45 programs (29%) that included a community event or gathering as part of the program. All events where the community was invited such as a camp, powwow, or fun run were categorized as community gatherings and events. One illustration of this is the Xeni Gwet’in First Nation’s youth programs in British Columbia. They have multiple youth-focused activities throughout the year such as camping, hunting, fishing, rafting, and horseback riding, as well as an annual eight-day trail ride that includes youth, families, and Elders [47,48]. The ride goes through traditional territory ending at the Williams Lake Stampede with the group camping together along the way.

#### 3.3.7. Spiritual Practices

Thirty-three programs (22%) described the inclusion of spiritual practices in their programming. The types of activities that were included in this category included prayer, smudging (burning of plant medicines), a sweat lodge, ceremonies, and other spiritual teachings. In one substance use prevention program out of Oklahoma for Cherokee youth and their parents, the primary land-based portion of the program was teaching participants to build a sweat lodge [49]. Additional examples of the incorporation of spirituality were programs starting or ending their day with prayer [50,51], making offerings [52], and smudging as they entered their gathering space [53].

#### 3.3.8. Talking Circles or Group Discussions

Thirty-one programs (20%) incorporated talking circles or a formal group discussion time into their program. One program for families in northern Ontario balances land-based cultural practices with time for “sharing circles” that gives participants the opportunity to talk about issues and tell stories [54]. The equine program Shonga Ska: Sacred Horse Society began each week of its eight-week program with a talking circle for youth to process their weekend [51]. Camp Pigaaq in Alaska closes its five-day camp with a talking circle [55].

#### 3.3.9. Significant Sites

Tribally significant sites were explicitly included in 28 programs (18%) and included locations such as traditional homelands, sacred or historical sites, and traditional hunting/fishing grounds. The Remember the Removal program developed by the Cherokee is an example of a program that includes significant sites, amongst other components [56,57,58]. This substance use prevention and wellness promotion program trains youth and young adults ages 16–24 in culture, history, language, and inherent Cherokee values alongside bicycling and physical exercise for five months. Next, the participants spend three weeks retracing the Trail of Tears on bike, stopping at significant sites to learn from cultural and historical experts.

#### 3.3.10. Formal Curriculum and/or Lessons

Programs (*n* = 21; 14%) were noted as having formal curriculum and/or lessons if they had and used a manualized curriculum that could be shared with or adapted by others. Nine of these twenty-one programs (43%) were community-derived, while another nine (43%) were developed by the community in collaboration with researchers. One originated from an outside organization, one was an adaptation of a program for the non-Indigenous population, and the last was developed by a community in collaboration with their local justice system. Several of the programs share materials online as a resource for other communities [59,60,61,62,63,64]. A few others offer training and/or curriculum for purchase [65,66,67].

#### 3.3.11. Animal-Involved

Nineteen programs (12%) involved animals—fourteen of which incorporated horses, and five included dog mushing. Eleven programs were based in the United States and seven in Canada. The Frank Attla Youth and Sled Dog Care-Mushing Program in Alaska’s primary focus was promoting wellbeing through involving youth with the cultural practice of dog mushing [68]. The “Our Life” program in New Mexico used an equine experience to promote healthy relationships between youth and parent participants [69].

#### 3.3.12. Storytelling

Seventeen programs (11%) included the oral tradition of storytelling, commonly by an Elder or other cultural knowledge keeper. For example, the Trails of our Ancestors program of the Tłı̨chǫ people in the Northwest Territories, Canada, is a 10–20-day canoe journey along significant sites for youth along the route; participants stop at locations such as old villages and graves where they are met by Elders who share stories of their ancestors. These stories convey, “all the knowledge necessary for living within the Tłı̨chǫ landscape” [70] (p. 29).

#### 3.3.13. Community Service

Sixteen programs (10%) incorporated some form of community service in their program. Some examples of service were community cleanups [46,71], the provision of food [43,44,72], and youth-selected service projects [24,39]. For instance, youth participants in the Aullak Sangilivallianginnatuk (Going Off, Growing Strong) Program in Newfoundland and Labrador fished, hunted, and prepared traditional foods to contribute to their community freezer and delivered food directly to Elders [72]. In the Northwest Territories, Wha Ti First Nation youth and young adults cleaned the trail to a popular berry gathering spot to provide their community with easier access [46].

#### 3.3.14. Language

Fourteen programs (9%) taught Indigenous language as a component of their activities. The Métis Settlements Life Skills Journey Program began as a summer day camp with life skill lessons developed through community-based participatory research between the University of Alberta and Métis settlements in the region [73]. Community leaders advocated for the integration of additional cultural knowledge, including Cree language, which was added into the programming on a site-specific basis [64].

#### 3.3.15. Celebrations and Feasts

Thirteen programs (8%) included a celebration and/or feast as part of their activities. One example of this is the Outdoor Adventure Leadership Experience in Ontario. This program features a 10-day canoe paddle through traditional territory, culminating in a welcome home feast with the community [74]. Another canoe-based program, the Mackenzie River Youth Leadership canoe trip, brings youth and Elders on a 16-day journey, with stops for ceremonies, celebrations, and feasts at each community along their route [75].

#### 3.3.16. Indigenous Values

Eleven programs (7%) specified that their traditional cultural values were imparted. At one week-long summer camp in the midwestern United States, the program activities integrated the seven values of Lakota life: (1) Woc’ekiya, prayer and communication with Tunkasila (the Creator); (2) Wa o’hola, respect for the self, higher power, community, and all life; (3) Wa on’sila, caring and compassion for others; (4) Wowijake, honesty and truth to one’s self and others; (5) Wawokiye, generosity without expecting anything in return; (6) Wah’wala, humility, we have a spirt, we are equals with others, and we are no better or less; and (7) Woksape, wisdom with experience comes from learning and practice [76]. The Hui Malama O Ke Kai afterschool program in Hawaii integrates five core cultural values into its program design and activities [71]. The values of aloha (love), mālama (to care for), ʻohana (family), kuleana (responsibility), and mahalo (gratitude, respect) are taught every day through experiential activities.

#### 3.3.17. Cultural Activities, Unspecified

A number of programs (55; 36%) indicated that participants were partaking in “cultural activities” but did not specify what those were. These were recorded in the data extraction process as “cultural activities, unspecified”. It is unclear if these are subsistence living activities, arts and crafts, or any number of possibilities.

#### 3.3.18. Additional Description of Outdoor and Land-Based Activities

Some programs were entirely outdoors, such as cultural camps, while others were largely in classrooms but with portions taking place on the land, such as a day spent picking berries. The land (and water)-based activities fell into four main categories: recreation, subsistence living, the visitation of significant sites, and animal-involved programming. The activities were diverse and reflected the landscapes, waterways, and cultural lifeways of the many different Indigenous nations. For example, the types of animals hunted and traditional foods prepared varied from moose and seal to birch syrup and bannock bread. Likewise, the recreational activities were environment-dependent, with some programs featuring warm weather activities like swimming and canoeing, while others in colder climates had ice skating and snowshoeing. For additional insights, Table 3 provides a list of the many varied activities that took place on the land as specified by the reviewed programs.

### 3.4. Evaluation Methods and Measures

There were 33 studies evaluating the outcomes of 28 land-based/outdoor programs for Indigenous youth and/or young adults. The studies incorporated a variety of designs including quantitative (9; 27%), qualitative (11; 33%), and mixed methods (13; 39%). Table S3 summarizes the studies, and their design, measured outcomes, instruments used, and findings. Amongst the quantitative and mixed method studies, a pre-/post-test design of program participants was commonly used. There were six studies that incorporated comparison groups [14,24,39,77,78,79], and six that were longitudinal [14,24,58,69,71,78]. The qualitative methods included interviews, focus groups, talking circles, observation, a document analysis, Photovoice, and “art voice”.

The quantitative and mixed method studies (*n* = 22) largely drew on existing instruments to measure their desired outcomes and sought tools that had been established as reliable and valid with Indigenous populations. Notably, many (9; 41%) either adapted instruments to fit their community [58,73,77,78,79,80,81], crafted supplemental questions around culture and community [79,82], or developed their own instruments to measure culturally appropriate outcomes [77,78,83,84]. A few incorporated existing scales developed for multicultural and/or Indigenous populations such as the Native American Enculturation Scale [69] and the Multigroup Ethnic Identity Measure [58,84]. One study compared the use of an Indigenous-developed instrument, the Aboriginal Children’s Health and Well-being Measure [28], to a previous study of their program using instruments developed for the general population [14] and found that it gave a more complete picture of the program effects. Two novel mixed method studies from Ahmed et al. [42,43] measured stress levels of land-based program participants through salivary cortisol levels. While the cortisol levels were either insignificant [43] or higher than expected after participation in a land-based activity [42], the qualitative findings revealed that participants perceived a number of benefits from the program.

Evaluation studies reported diverse, positive outcomes of programs that largely align with their stated aims of connection to culture, community, the environment, and personal development. The most common outcomes included personal development, connections to culture, and connections to community.

#### 3.4.1. Personal Development

Six studies reported positive impacts on self-esteem [68,69,71,73,82,84] and three reported increases in self-confidence [57,80,85]. Positive mental health impacts were described by six studies [14,28,45,58,80,86]. An increased sense of wellbeing was mentioned by four studies [42,43,44,87]. Three reported increases in resilience [14,68,84], while two described positive impacts on coping/ability to deal with life’s stressors [39,55]. Lastly, two studies expressed improvements in emotional health [28,80], two described improvements in mood [55,80], two studies saw increases in hope [39,68], and three found reductions in depression [39,58,82].

#### 3.4.2. Connection to Culture

Nine programs reported outcomes related to increased connection to culture [14,42,43,44,68,80,85,88,89]. Two described increases in cultural pride [68,81] and three reported increased cultural identity [58,69,84]. Five described increased knowledge and practice of culture/language/values [58,71,82,83,90].

#### 3.4.3. Connection to Community

Several programs described positive impacts on the social connections of participants. This ranged from increased connection to community reported in seven studies [14,44,68,80,87,90,91] to connection to peers found by four studies [14,68,80,90], connection to Elders specified by four studies [14,42,68,82], the sharing of intergenerational knowledge described in three studies [44,45,88], and connection to ancestors reported by three studies [14,88,90]. Three studies relayed impacts on family-related outcomes, including family cohesion [71], family bonds [87], and connection with family [90]. Social connections extended to positive effects on sense of belonging found in three studies [55,68,85] and social support reported by two [58,91].

#### 3.4.4. Connection to Environment

Participants reported the importance of their connection to the environment and program impacts on this outcome in qualitative findings of eight studies [14,41,42,45,49,87,88,90], but this was not a measured outcome in quantitative studies.

#### 3.4.5. Other

Five programs listed positive effects on the physical health of participants [28,45,58,68,80]. Four measured improved outcomes related to spiritual health [28,80], described also as connection to spirituality [87] and connection to the Creator [14]. Five programs demonstrated positive effects around substance use-related outcomes [24,71,73,79,82]. Lastly, two studies reported outcomes related to leadership skills [85,89].

## 4. Discussion

The purpose of this scoping review was to explore the existing literature on outdoor and land-based prevention programs for Indigenous youth and young adults, and to characterize their design, aims, evaluation methods, and outcomes. These programs appear to have widespread acceptance within Indigenous communities and most often originate from the communities themselves. Cultural teachings and lifeways feature predominately, with the involvement of Elders being key as holders of that cultural knowledge. Although many programs have not undergone formal evaluation, there is a substantial number demonstrating positive outcomes on the health and wellbeing of their participants, especially personal, cultural, and relational growth. Multiple communities have partnered with researchers to evaluate their outdoor and land-based programs, and there is growing evidence of their positive impacts on participants, including improved mental health, increased self-esteem, and increased connections of participants to their culture and community.

When Indigenous communities create their own behavioral health programs, they are often land-based [33]. Nearly 90% of programs included in this review originated from Indigenous communities, including the portion in collaboration with institutional researchers. There are over 1200 unique Indigenous nations throughout North America [92,93], and several hundred more when you include Tribes in the United States without federal recognition [94]. Programs that derive from these numerous communities would understandably be as diverse as the peoples and places they stem from. Given that very diverse landscape, however, this review revealed that there are some common threads weaving through outdoor and land-based programs for Indigenous youth.

The single most pervasive activity of the reviewed programs was engagement in cultural lifeways, which is consistent with characterizations of land-based programs as being grounded in Indigenous knowledge and ways of life and incorporating cultural teachings and practices [3,4]. Land is understood to be a source of Indigenous cultures, and traditional lifeways are inextricably tied to the local environment. Almost all of the programs included cultural knowledge and/or practices, even those programs that exhibited more features of an outdoor adventure model. Participation in cultural activities has the potential to simultaneously strengthen youth’s self-concept and sense of belonging to an Indigenous community, while increasing their cultural knowledge and connection. Developing a sense of identity is one of the key tasks of adolescence and much research has been conducted in this area [95]. For young people of color, a positive cultural identity promotes healthy development and may additionally be protective against negative messages and discrimination youth experience in society [96]. Connection to culture has been shown to have positive associations with mental health for Indigenous youth [97,98] and cultural identity has been associated with reduced risk of substance use for Native youth and young adults [99]. In this review, the outcome-based studies indicated that participants reported significant improvements in self-esteem and self-confidence, mental and emotional health, and growth in their connection to and knowledge of their culture and cultural identity. One land-based program participant described how this operates.

“I was happy that I got to learn who I was as an Aboriginal youth. I used to be so ashamed of being Native and now I’m not … so yeah, it made me happy for who I am and where I come from” [82] (p. 27).

After this inherent cultural foundation, the most common activities of the outdoor and land-based programs in this review were categorized as recreation and subsistence living. These activities took place on the land or water and often included cultural practices and knowledge. “Recreational” activities were oftentimes traditional modes of transportation (e.g., canoeing, snowshoeing, hiking, horseback riding) or activities such as Indigenous games or archery, but also included contemporary outdoor recreation such as snowboarding, skiing, and rafting. Subsistence living activities reflected the traditional land- or water-based practices common to the area, for example, fishing, hunting, traditional ecological knowledge, shelter building, and survival skills. Oftentimes, communities held multiple camps based on seasonal activities. The on-the-land setting of these activities may provide unique health benefits. Connection to the land has been articulated as a social determinant of health by Indigenous youth [100], and many of the qualitative studies in this review likewise described the necessity of connecting to the environment for wellbeing. Time spent in nature has been shown to decrease stress, improve mood, increase attention span, and promote physical activity in youth [7,101]. One land-based program developer shared the following:

“I struggled with alcohol as a young man and one of- one of the biggest things that helped me was going out, with my family, out on the land. It was like going to a treatment centre. You felt good. You woke up good, you know when you sleep on the ground it’s like you’re grounding yourself again. You wake up, you feel just energized, you feel good, happy to be alive” [54] (p. 5).

Many of the Indigenous lifeways youth participated in during the programs are regarded as recreation or leisure activities today, so it is perhaps not surprising that they produce the physical and psychological effects of such activities. The subsistence living practices many program participants engaged in such as hunting, fishing, and picking berries are activities that also naturally promote physical activity [80]. Several of the programs in this review found not only mental and behavioral health benefits, but also positive impacts on physical activity and nutrition. Exposing youth to the outdoors and ancestral lifeways could counteract the negative health impacts of our increasingly sedentary lifestyle.

Connections to family, friends, caring adults, and the community as a whole are all protective factors for Indigenous youth [97]. Many of the programs in this review aimed to increase these social supports, as well as a sense of belonging, and experienced success in doing so. Programs were group-based, at times including families or community members, and if not in the regular activities, then in special gatherings or events. Intergenerational connections were commonly fostered through Elders and knowledge keepers serving as instructors. Elders or other cultural knowledge keepers were frequently utilized to share their wisdom of cultural practices, teachings, and values with the younger generations. The involvement of Elders has often been described as an essential element of land-based programs [3,102,103]. The studies found that participants experienced increases in connections to community, peers, and Elders, and to a somewhat lesser degree, their family and ancestors.

Several studies included in this review measured holistic wellbeing outcomes that included not only mental, emotional, social, and physical health, but also spiritual health. Their positive findings were supported by qualitative studies where participants perceived programs as having holistic benefits on wellbeing, including spirituality. For example, one youth participant in a land-based program shared, “I do feel a change like I feel that my spirit is fed … like with goodness” [45] (p. 12). This aligns with another systematic review of land-based programs for Indigenous adults where participants also reported benefits inclusive of mental, emotional, social, physical, and spiritual health [34]. Multiple programs and land-based practitioners described their ultimate desired outcome as connecting youth to what translated to “the good life”, or a healthy way of living as defined by their community [4,41,52]. While the researchers were oftentimes able to utilize existing, validated instruments to measure outcomes, close to half made cultural adaptations or created their own mechanisms to more accurately measure outcomes of interest, especially around culture, community, and holistic wellbeing. As noted by Usuba, et al. [28], while it is possible to measure effectiveness using instruments developed and validated on other populations, it is best to use evaluation tools that reflect the culture and values of the intended community. A more holistic evaluation designed for Indigenous populations and including measures of mental, emotional, social, physical, and spiritual health as well as environmental connections may be needed to capture the complete impacts of outdoor and land-based programs.

### Limitations

Although our search strategy was designed to be comprehensive and inclusive, there are undoubtedly programs in the literature that were not captured in this scoping review. In addition, there were challenges in deciding how to define outdoor or land-based programs, such as is there a certain percentage of time that needs to be outdoors? We chose to lean toward inclusivity in order to gather the broadest picture possible, and included programs that intentionally provided any elements that took place on the land. The inclusion of the grey literature greatly increased the amount of the literature and number of programs identified in the search. However, the descriptions of programs in government reports especially were brief and may not have fully articulated all components of the programs. We did our best to fill in the gaps with further web searches to find additional program information, where available. The number of peer-reviewed studies on outdoor and land-based programs has grown significantly in recent years, as documented by this review. Prevention programs inclusive of both land and culture are showing some early indications of effectiveness for Indigenous youth. Study quality was not assessed in this paper, so findings must be interpreted cautiously. These studies were not all experimental designs, and a few were longitudinal. Future research should examine the quality of evidence for outdoor and land-based programs. Additionally, researchers must continue to grapple with what constitutes rigorous evidence, with respect for both Western evidence and Indigenous ways of knowing.

## 5. Conclusions

The literature on outdoor and land-based prevention programs for Indigenous youth is growing rapidly. Across diverse Indigenous communities, some common features of these programs include cultural lifeways, recreation, subsistence living, and Elder knowledge sharing. A number of communities have partnered with researchers to examine the impact of their programs and found positive effects on mental health, self-esteem, social connections, and cultural identity. The research indicates that there are likely additional benefits to physical and spiritual health, and connections to the environment that have not always been measured. There are several programs with formal curriculum that other Indigenous communities could learn from when developing their own programs. Understanding the components of such programs is a first step to identifying the elements critical to effective outdoor and land-based programs for Indigenous youth.

## Figures and Tables

**Table 1:** Literature types.

Literature Type	Number
Peer-reviewed articles	51
Reports	25
Websites	8
Newsletters	4
Theses	4
Videos	2
Conference proceedings	2
PowerPoint presentations	1
Pre-print articles	1
TOTAL	98

**Table 2:** Activities by number of programs in which they were found.

Activities	No. of Programs with This Activity
Recreation	114 (75%)
Subsistence Living Skills	100 (65%)
Elder Knowledge Sharing	97 (63%)
Cultural Activities, Unspecified	55 (36%)
Arts and Crafts	54 (35%)
Community Events and Gatherings	45 (29%)
Spiritual Practices	33 (22%)
Talking Circles or Group Discussions	31 (20%)
Visits to Significant Sites	28 (18%)
Formal Curriculum and/or Lessons	21 (14%)
Animal-Involved	19 (12%)
Storytelling	17 (11%)
Community Service	16 (10%)
Indigenous Language	14 (9%)
Celebrations and Feasts	13 (8%)
Tribal-Specific Values	11 (7%)

Note. One program can contain multiple activities.

**Table 3:** Specific on-the-land activities and numbers of programs including them.

Recreation (128 Programs)
Traditional: camping (75), canoeing (28), hiking/walking/trek (21), traditional games/sports (13), swimming (11), snowshoeing (10), running (8), archery (6), kayaking (3) Contemporary: boating (9), rafting (6), snowmobiling (5), skiing (3), ice skating (3), hockey (3), firearm safety (3), snowboarding (2), bicycling (2), rock climbing (2), high-ropes course (1), ice golf (1), rifle shooting (1)
**Subsistence Living (100 programs)**
Hunting (51), fishing (39), and trapping (28), including whale, moose, caribou, walrus, seal, beaver, goose, duck, muskrat, fish, and clam digging Camp/shelter building (9), igloo construction (3), tipi set-up (1), qammaq/winter house construction (1), waashaaukimikw/dwelling construction (1) Plant knowledge (14), medicines (15), berry picking (10), gardening (4) Traditional food preparation (25) included dry or smoked meat/fish, birch syrup, bannock bread, egg picking, edible plants, and teas Survival skills (17), tool making (7), fire building (7), navigation (4), water safety (4), animal tracking (3), ice safety (1), traditional weather forecasting (1) Hide tanning (6)
**Significant Sites (28 programs)**
Types of significant sites described: historical sites (13), sacred sites (7), traditional homelands (6), traditional hunting/fishing/gathering grounds (5), and other places of cultural significance (4)
**Animal-Involved (19 programs)**
Equine experience (14), dog mushing (5)

Note. One program can contain multiple activities or have visited multiple significant sites.

## Data Availability

The original contributions presented in this study are included in the article/Supplementary Materials. Further inquiries can be directed to the corresponding author.

## Supplementary Materials

The following supporting information can be downloaded at: https://www.mdpi.com/article/10.3390/ijerph22020183/s1, Table S1: Sample search strategy used for PubMed database, Table S2: Data extraction form, and Table S3: Outcome evaluations of outdoor and land-based programs.
