# Supplementary material for: Scoping Review of Outdoor and Land-Based Prevention Programs for Indigenous Youth in the United States and Canada
Source: Int J Environ Res Public Health. 2025 Jan 28;22(2):183. doi: 10.3390/ijerph22020183 (PMC11855302; doi:10.3390/ijerph22020183)
Supplement: Supplementary file 1 [file ijerph-22-00183-s001.zip › ijerph-3359382-supplementary.pdf]

Table S1. Sample search strategy used for PubMed database.*

Database	Filters Applied	Boolean Phrase
**PubMed**	Adolescence, Young Adult 18-24, Child 6-12, publication date 2000-2022	*("Native American" OR "American Indian" OR "Tribal" OR "Indigenous" OR "First Nations") AND ("land-based" OR "land based" OR "outdoor" OR "adventure" OR "wilderness" OR "recreation" OR "cultur*" OR "equine") AND ("program" OR "intervention" OR "prevention" OR "camp") AND (outcome OR evaluation OR impact OR effectiveness)*

*An academic librarian provided consultation on the search strategy.

Table S2. Data extraction form.

**General Information**	
Study ID:	
Title:	
Authors:	
Year of Publication:	
Country in which the study was conducted:	¨ United States<br>¨ Canada<br>¨ Other:
**Characteristics of Intervention**	
Tribal Community (if disclosed include Tribal Nation(s), reservation/reserve, and/or city/state/province):	
Intervention Name:	
Type of Intervention	¨ Suicide Prevention<br>¨ Substance Use Prevention<br>¨ Wellness Promotion<br>¨ Other:
Intervention Origination/Development	¨ Adopted as is<br>¨ Adaptation<br>¨ Community-derived<br>¨ Not specified<br>¨ Other:
Target Population Description (specify youth, young adults, families, age range)	
Duration of the Intervention (i.e. dosage: one time event, week-long camp, 10 one-hour lessons, once/week for a full-year, etc.)	
Aim of Intervention (i.e. intended outcomes like reduce depression, decrease substance use, etc.)	
Intervention Components (check all that apply):	¨ Formal curriculum and/or lessons<br>¨ Community events and gatherings<br>¨ Elder knowledge sharing<br>¨ Smudge, sweat, ceremony<br>¨ Celebrations, feasts

	¨ Talking circles or group discussions<br>¨ Arts and crafts<br>¨ Subsistence living skills<br>¨ Recreation<br>¨ Equine experience<br>¨ Community service<br>¨ Cultural activities, unspecified<br>¨ Other:
Specific Land-based or Outdoor Activities:	
**Evaluation**	
Study Design	¨ Randomized controlled trial<br>¨ Non-randomized experimental study<br>¨ Qualitative research<br>¨ Program report<br>¨ No evaluation conducted<br>¨ Other:
Findings/Results (if quantitative study done, list outcomes measured by name, and specify findings i.e. X increase in resiliency):	

Table S3: Outcome evaluations of outdoor and land-based programs.

Citation	Program	Location	Study design	Outcome Measures/ Measurement Instruments	Findings
Quantitative Studies					
Allen, et al. (2023)	Qungasvik	Yup'ik communities<br><br>Alaska, United States	Quantitative<br><br>Dynamic wait-listed design of four communities and with four measurement time points - twice prior to intervention, once midway through intervention, and once two years following intervention	Yup'ik culture-specific protective factors: Elluarrluni piyugngariluni "Learning in the mind of doing things in a masterful way," Elluarrluteng ilakelriit "Nurturing family," Nunamta "Our community," Umyuangcaryaraq "Reflecting" (Reflective Processes), Yuuyaraqegtaar "A way to live a very good, beautiful life" (Reasons for Life)<br><br>Multicultural Mastery Scale, Brief Family Relationship Scale, adapted Yup'ik Protective Factors Scale, adapted Brief Reasons	The intervention was associated with dose dependent growth in long-term outcome variables of "reasons for life" and "reflective processes on alcohol consequences," but not intermediate variables.

				for Living Inventory for Adolescents	
Barnett, et al. (2020)	Camps Pigaaq and Igalik	Iñupiaq and Yup'ik communities<br><br>Alaska, United States	Quantitative<br><br>One group, pre-/post design	Self-esteem, emotional states, belongingness, mattering to others, and coping skills<br><br>International Positive and Negative Affect Schedule–Short-Form, Multicultural Mastery Scale, Interpersonal Needs Questionnaire (belongingness subscale), General Mattering Scale, Rosenburg Self-Esteem Scale	Participants reported more positive mood, an increased sense of belongingness, and greater perceived internal ability to handle potential life stressors. No significant changes for participants' perception of mattering to others, self-esteem, or perceived support for coping with life stressors from friends or family
Carter, et al. (2007)	Project Venture	Tribe(s) unspecified<br><br>New Mexico, United States	Quantitative<br><br>Quasi-experimental longitudinal design with treatment and control groups	Past 30-day alcohol use, and three other substance use measures not provided	Found a significant difference between the substance use patterns of treatment and control participants across time, with treatment youth demonstrating less growth in substance use.
Hibbert, et al. (2018)	Métis Settlements Life Skills Journey Program	Buffalo Lake, Kikino, Fishing Lake, Elizabeth, East Prairie, Gift Lake,	Quantitative<br><br>One group pre-/post survey design	Resilience<br><br>Youth Resiliency: Assessing Developmental Strengths Questionnaire (adapted)	Positive change occurred in several areas of internal strength, including self-esteem, drug resistance, and planning and decision making.

		and Peavine Métis Settlements<br><br>Alberta, Canada			
Hishinuma, et al. (2009)	Hui Malama O Ke Kai (The Caring Group from the Ocean)	Native Hawaiians<br><br>Hawaii, United States	Quantitative<br><br>Cross-sequential pre-/post-test design across three program years	Family cohesion, Native Hawaiian values, Native Hawaiian pride, school success, self-esteem, antidrug use, violence prevention strategies, and healthy lifestyle<br><br>AmeriCorp Achieve Impact Survey	Findings for Year 1 included: significant increases in knowledge and practice of Native Hawaiian cultural values for youths and for parents' perception of their child's knowledge and practice of cultural values. In addition, increases in youth self-esteem, antidrug use, violence prevention strategies, and healthy lifestyle, as well as parents' perception of family cohesion and school success. Findings for Year 2 included: increases in youths' perceived family cohesion, school success, and both parent and youths' perceived violence prevention strategies.
Lewis, et al. (2022)	Remember the Removal Program	Cherokee Nation (Oklahoma) and Eastern Band of Cherokee Indians	Quantitative<br><br>One-group, longitudinal design. Completed surveys four times: before the program, at end of the training period, at program end,	Physical health: body mass index, diet, food intake, physical activity, self-efficacy for activity; mental health: positive mental health, post-traumatic stress disorder, stress, depression,	At program end, participants saw statistically significant improvement in diet and exercise, reduced stress, anxiety, depression, anger, post-traumatic stress disorder, and microaggressions, and improved positive mental health, social

(North Carolina)<br><br>United States	and a six-month follow-up	anxiety, anger, microaggressions, historical trauma; social/cultural health: social support, Cherokee identity, Cherokee language, Cherokee traditional ways, community values<br><br>International Physical Activity Questionnaire-Short Form, Self-Efficacy for Activity Scale, Mental Health Continuum—Short Form, Primary Care PTSD Screen, Global Stress Assessment, Patient Health Questionnaire-9, Generalized Anxiety Disorder 7-Item Scale, Tri-Ethnic Anger Scale, Microaggressions Measure (adapted version), Historical Loss and Associated Symptoms, Social Support Survey, Multigroup Ethnic Identity Measure, Cherokee Nation Community Values	support, Cherokee identity, and Cherokee values. At the six-month follow-up, participants maintained improvements in depression, anger, microagressions, and Cherokee identity.

Redvers, et al. (2021)	Arctic Indigenous Wellness Foundation Urban Land-Based Healing Camp	Urban Indigenous community<br><br>Northwest Territories, Canada	Quantitative<br><br>One group pre-/post design	Emotional state	Results indicated there was a statistically significant difference in how participants were feeling pre- and post-program, with the majority reporting positive feelings at the end of camp.
Usuba, et al. (2019)	Outdoor Adventure Leadership Experience	Anishinaabe of Wikwemikong Unceded Indian Reserve<br><br>Ontario, Canada	Quantitative<br><br>One-group pre-/post design	Spiritual, emotional, physical and mental health<br><br>Aboriginal Children's Heath and Well-being Measure (ACHWM), Global Health Rating (GHR)	Participant scores in all four quadrants (spiritual, emotional, physical, and mental) of the ACHWM improved between pre- and post-program, and there was a statistically significant difference in their overall ACHWM score. More youth reported a better GHR score post-program as well, however this was not statistically significant.
Qualitative Studies					
Ahmed, et al. (2023)	Sibi (River) Program	Fort Albany First Nation Omushkego Cree<br><br>Ontario, Canada	Qualitative<br><br>Photovoice and semi-structured interviews	N/A	Enhanced well-being attributed to the promotion of social and community networks, sharing of intergenerational knowledge, and advancement of cultural continuity.
Barwin, et al. (2013)	Traditional Medicine Programming	Ojibway, Pottawatomi and Odawa, and	Qualitative<br><br>"Art Voice," focus groups, and interviews	N/A	Results indicated the need to approach traditional teachings, health programs, and research from an Indigenous worldview,

		Anishnawbek of Manitou Minissing descent<br><br>Ontario, Canada			and that more frequent workshops are required to empower both youth and adults to practice and share traditional knowledge. In addition, interest in language, culture, and tradition increased with age.
Gaudet, et al. (2018)	Milo Pimatisiwin Project	Moose Cree First Nation<br><br>Ontario, Canada	Qualitative<br><br>Visiting, conversations, observation, and experiential learning	N/A	Three themes that emerged were: 1) outsiders such as researchers need to learn from and with the community, 2) Indigenous view of health and wellbeing includes relationships with land, identity, and family, 3) Elder involvement is essential in land-based programs.
Hickey, et al. (2020)	Family Camping Weekend	Urban Indigenous community<br><br>New Brunswick, Canada	Qualitative<br><br>Interviews, research circle using talking stick, document review (journals)	N/A	Themes included: relationship and connection to land, connection to spirituality, strengthened bonds between family and community, sense of wellbeing and empowerment.
Lewis, et al. (2020)	Remember the Removal Program	Cherokee Nation (Oklahoma) and Eastern Band of Cherokee Indians	Qualitative<br><br>Focus groups	N/A	Participants shared what they learned or gained from the program. Five themes emerged: treat everyone with kindness, help each other and work together, take care of one another, treat each other as family, and be confident or self-efficacy.

		(North Carolina)<br><br>United States			
Mikraszewicz and Richmond (2019)	Biigtig Canoe Journey	Biigtigong Nishnaabeg<br><br>Ontario, Canada	Qualitative<br><br>Semi-structured interviews	N/A	Analysis of the interview data led to three findings regarding the program. It was a space to: 1) learn and practice Indigenous Knowledge; 2) connect with other participants, family, community, ancestors, and the river, 3) connect to the land of their ancestors and as a source of Indigenous lifeways.
Philip, et al. (2022)	Frank Attla Youth and Sled Dog Care-Mushing Program	mostly Athabascan community<br><br>Alaska, United States	Qualitative<br><br>Photovoice, digital storytelling, focus groups	N/A	The program strengthened youth participant connections and trusting relationships with Elders, adults, other youth, animals, and the community. Participants discussed an increased connection to culture and sense of belonging and pride in their community and cultural identity.<br>Youth discussed how the program built self-esteem, discipline, respect, resilience, feelings of hope and optimism and promoted physical activity.
Ritchie, et al. (2015)	Outdoor Adventure	Anishinaabe of	Qualitative	N/A	Participants reported connecting with creation (including: Mother

	Leadership Experience	Wikwemikong Unceded Indian Reserve<br><br>Ontario, Canada	Interviews, journals, focus groups, and talking circles		Earth, people, community and culture, ancestors and Elders, plants and animals, and Creator) and connecting with self (physically, mentally, spiritually, emotionally, and holistically) and their identity. Ultimately, the program helped them connect to Anishinaabe Bimaadziwin (the Good Life).
Robbins, et al. (2016)	The Gifts of the Seven Directions	Majority Cherokee<br><br>Oklahoma, United States	Qualitative<br><br>Written responses to open-ended questions	N/A	Common themes reported were participants feeling less alone (universality), learning about themselves through group interactions, valuing of group cohesion, connecting to nature, and appreciating Indian humor as part of the program experience
Takano (2005)	Paariaqtuqtut (Meeting on the Trail)	Inuit<br><br>Nunavut, Canada	Qualitative<br><br>Primarily participant observation and semi-structured interviews, supported by document analysis	N/A	The community saw the program as necessary because young people were not learning traditional lifeways due to breakdown of traditional modes of knowledge transfer. They felt efforts needed to be made to preserve and promote Inuit culture, and teach a better way of life that could prevent social problems. Subsistence skills provided food, both as an economic resource and cultural practice. Connection to the land

					was seen as an essential part of being Inuit. Young participants in the program shared they would like to be on the land more, learn more culture, and get a break from town life. They reported learning mostly practical things, and connecting with their ancestors, culture, and the land.
Walker, et al. (2021)	Smart Indigenous Youth	Cree and Saulteaux<br><br>Saskatchewan, Canada	Qualitative<br><br>Pre-/post intervention focus groups	N/A	Overall focus group analyses revealed four main themes: Indigenous culture, identity, mental health and physical activity. Post-intervention youth expressed an increased interest in retaining culture and identity, respect and gratitude for the land, improved self-concept, mental health, and physical activity, and the importance of intergenerational learning from Elders to younger generations.
Mixed Methods Studies					
Ahmed, et al. (2023)	Niska (Goose) Program	Fort Albany First Nation Omushkego Cree<br><br>Ontario, Canada	Mixed methods<br><br>Quant: one group pre-/post design<br><br>Qual: Photovoice and semi-directed interviews	Salivary cortisol levels (biometric measure of stress)	Increase in subjective well-being. Provided opportunity to connect with identity and increase social cohesion and well-being within the community.<br><br>Changes in cortisol levels were not statistically significant.

Ahmed, et al. (2022)	Amisk (Beaver) Program	Fort Albany First Nation Omushkego Cree<br><br>Ontario, Canada	Mixed methods<br><br>Quant: one group pre-/post design<br><br>Qual: Photovoice and semi-directed interviews	Salivary cortisol levels (biometric measure of stress)	Increase in subjective well-being. Reconnected youth with their traditional homeland, their Elders, and cultural traditions.<br><br>No significant changes in cortisol levels were observed in the participants post-trapping, and a statistically significant increase was observed post-removal of the beaver dams.
Crooks, et al. (2015)	Fourth R: Uniting our Nations	Chippewas of the Thames First Nation, Oneida Nation of the Thames, and Munsee-Delaware First Nation<br><br>Ontario, Canada	Mixed methods<br><br>Quant: one-group post-test only survey data<br><br>Qual: interviews	Student success, relationships and belonging	Reported increase in student success, sense of belonging, confidence and leadership skills, and connection to culture especially for those students who attended culture camp which was the land-based element of the program.
Harder, et al. (2015)	Nges Siy (I love you)	11 Carrier and Sekani Nations<br><br>British Columbia, Canada	Mixed methods<br><br>Quant: one-group pre-/post design<br><br>Qual: interviews and focus groups	Symptoms of depression, suicidal ideation, hopelessness, self-esteem<br><br>Beck Depression Inventory-II, Beck Scale for Suicide Ideation, Beck	Results indicated that levels of depression and suicidal ideation significantly decreased, and self-esteem significantly increased after youth participated in the intervention. Survey results reflected increases in knowledge of language, culture and

				Hopelessness Scale, Rosenberg Self-Esteem Scale, and an additional survey that asked questions around cultural identity, connection to community, and risk-taking behavior.	connection to Elders, and youth expressed a reduced desire to use substances.<br><br>Themes from qualitative analysis included: 1) cultural awareness, connection, and identity; 2) self-awareness, enhancement, and discovery; and 3) attitudinal, behavioural, and developmental changes
Goodkind, et al. (2012)	Our Life	Tribe(s) unspecified<br><br>New Mexico, United States	Mixed methods<br><br>Quant: quasi-experimental one-group longitudinal design, with five measurement points – baseline, midway through intervention, end of intervention, 6 and 12 months post-treatment<br><br>Qual: interviews	Exposure to violence, PTSD symptoms, enculturation, self-esteem, coping, quality of life, social functioning<br><br>Life Events Scale, Childhood PTSD Symptom Scale, Native American Enculturation Scale, Harter Self-Perception Profile for Children, Rosenberg Self-Esteem Scale, Children's Coping Strategies Checklist, Multidimensional Student's Life Satisfaction Scale, Social Adjustment Inventory for Children and Adolescents	Youth who completed at least 9 intervention sessions, experienced significant increases in their traditional cultural identity, self-esteem, positive coping strategies, quality of life, and social adjustment.<br><br>Qualitative findings supported quantitative results and suggested additional positive effects.

Healey, et al. (2016); Mearns and Healey (2015)	Makimautiksat Youth Wellness and Empowerment Camp	five Inuit communities<br><br>Nunavut, Canada	Mixed methods<br><br>Quant: one-group pre-/post design<br><br>Qual: focus groups and observation	Evaluation questionnaire adapted from Keyes Mental Health Continuum Short Form Questionnaire, SHAPES Mental Fitness Module, Qanuippitali Inuit Health Survey, and program specific questions.	Findings indicated the program fostered physical, mental, emotional, and spiritual wellness. Participation increased connection to community, peers, and culture. Participants and parents reported improved mood, attitude, and self-confidence.
Hunter, et al. (2022)	Native Spirit	Urban-based reservations - Akimel O'odham (River People) and the Xa-lychidom Piipaash (People Who Live Toward the Water)<br><br>Arizona, United States	Mixed methods<br><br>Quant: one-group pre-/post design<br><br>Qual: interviews	Cultural identity, self-esteem, and resilience<br><br>Multigroup Ethnic Identity Measure, Rosenberg Self-Esteem Scale, Child and Youth Resilience Measure	Results showed post-program increases in resilience and self-esteem and a statistically significant increase in cultural identity.<br><br>In qualitative analysis, participants expressed interest in learning more about their cultural identity and more confidence in their cultural knowledge, as well as positive effects on self-esteem and resilience.
Janelle, et al. (2009)	Atikamek Wilderness Project	Atikamek community of Manawa	Mixed methods<br><br>Quant: quasi-experimental one-group post-test-only design	Self-esteem, pro-social behavior, cultural pride, cultural promotion, and community mobilization	The analysis of the systematic observation suggested that participants were motivated to engage in cultural practices and exhibited cooperative and pro-

		Quebec, Canada	Qual: participatory observation using an observation grid	Adaptations of State Self-Esteem Scale, Rosenberg Self-Esteem Scale	social behaviors, cultural pride, and pride in accomplishments during the program.<br><br>Quantitative portion had no reported results as scales were discarded after being perceived as problematic to participants.
Kelley, et al. (2018)	Culturally-based Prevention Activities	five reservation communities and one urban Indian community<br><br>Rocky Mountain Region, United States	Mixed methods<br><br>Quant: post-test-only design with surveys of intervention and non-intervention youth<br><br>Qual: interviews for community readiness assessment portion of study	Drug and alcohol use, culture, community connections, social support, self-esteem, family communications<br><br>Questions selected or adapted from: Youth Risk Factor Behavior Survey Standard Questionnaire, Multidimensional Scale of Perceived Social Support, Rosenberg Self-Esteem Scale, and questions on culture and community connections developed by community members	Scores for social support, community connections, and family communication about substance use were significantly higher among intervention youth than non-intervention youth. No significant difference was found for substance use between the two groups.
Ritchie, et al. (2014)	Outdoor Adventure Leadership Experience	Anishinaabe of Wikwemikong Unceded	Mixed methods<br><br>Quant: questionnaire administered to treatment and	Resilience, mental health<br><br>Resilience Scale, Mental and Physical component scores from the Short	Resilience scores improved significantly between baseline and one month after the intervention, but then reverted back to pre-test levels one year post-intervention.

		Indian Reserve<br><br>Ontario, Canada	comparison group 1-day before intervention, 1 month after, and one year after<br><br>Qual: Open-ended structured questions administered one year post-intervention	Form 12v2 Health Survey, Scale of Positive and Negative Emotion, Flourishing Scale, Self-Esteem Scale, Satisfaction with Life Scale, social support and spiritual values from the Canadian Community Health Survey, community values questions from the Child and Youth Resilience Measure	Mental health scores continued to improve over the three time periods. All other wellbeing scales showed a positive change between baseline and one-month post-intervention, but with varied results at one year.
Sy, et al. (2015)	Hui Malama O Ke Kai (The Caring Group from the Ocean)	Native Hawaiians<br><br>Hawaii, United States	Mixed methods<br><br>Quant: within-group pre-post design<br><br>Qual: interviews, journals, retreat evaluations	Child's Hawaiian values<br><br>Hui Malama O Ke Kai Rubrics of Hawaiian Values, General Learner Objectives from Hawaii Department of Education	Participants had significant increases in the primary outcome of interest - their understanding and practice of Hawaiian values.<br><br>Quantitative and qualitative data sources corroborated each other and indicated the different data types, and the Hui Malama O Ke Kai Rubrics of Hawaiian Values instrument, were valid and reliable.
Tosa, et al. (2018)	Reducing Risk through Interpersonal Development, Empowerment, Resiliency, and	Pueblo of Jemez<br><br>New Mexico, United States	Mixed methods<br><br>Quant: pre-/post design with intervention and non-participant groups	Coping, gratitude, optimism, empowerment, self and collective efficacy, depression	For those participants with high attendance (exposure), there was a significant difference in their scores for hope pre- and post-program. There was also significant difference between

	Self-Determination (RezRIDERS)		Qual: semi-structured interviews	symptomology and alcohol use<br><br>Brief Cope Scale, Life Orientation Test-Revised, Hope Scale, Gratitude Questionnaire, Wiggins Empowerment, New Mexico Youth Risk and Resiliency Surveillance Survey	youth with high exposure and the control group on depression and coping.<br><br>Parent interviews revealed two themes: youth improvement in "decision-making" and increased "self-expression" or communication.
Trousdale, et al. (2017)	Supporting Emerging Aboriginal Stewards (SEAS) Community Initiative	Bella Bella (Heiltsuk Nation), Klemtu (Kitasoo Xai,Äôxais Nation) and Bella Coola (Nuxalk Nation)<br><br>British Columbia, Canada	Mixed methods<br><br>Quant: one-group post-test only design<br><br>Qual: interviews and focus groups	Not provided	Findings suggest positive impacts for youth in: leadership, school performance, character development, opportunity, connection to territory and culture, and health and wellness. Youth and educators are most consistently reporting the following outcomes:<br>1. Passionate about caring for the lands and waters, living things<br>2. Concerned about others' wellbeing (compassion)<br>3. Connected to their Nation's places (e.g., sites of cultural and ecological importance)<br>4. Respectful (of others, nature)<br>5. Connected to their Nation's ways (e.g., stories, laws, practices, values)<br>6. Mentally supported by their

connection to the outdoors
7. Enthusiastic about learning and achieving
8. Comfortable with new people and ideas
9. Open to new challenges
10. Able to retain information and teachings
